# β2-Adrenergic Receptor Knockout Mice Exhibit A Diabetic Retinopathy Phenotype

**DOI:** 10.1371/journal.pone.0070555

**Published:** 2013-07-24

**Authors:** Youde Jiang, Qiuhua Zhang, Li Liu, Jie Tang, Timothy S. Kern, Jena J. Steinle

**Affiliations:** 1 Department of Ophthalmology, University of Tennessee Health Science Center, Memphis, Tennessee, United States of America; 2 Department of Anatomy and Neurobiology, University of Tennessee Health Science Center, Memphis, Tennessee, United States of America; 3 Department of Ophthalmology and Medicine, Case Western Reserve University, Cleveland, Ohio, United States of America; 4 Department of Research, Stokes Veterans Administration Hospital, Cleveland, Ohio, United States of America; Cedars-Sinai Medical Center, United States of America

## Abstract

There is considerable evidence from our lab and others for a functional link between β-adrenergic receptor and insulin receptor signaling pathways in retina. Furthermore, we hypothesize that this link may contribute to lesions similar to diabetic retinopathy in that the loss of adrenergic input observed in diabetic retinopathy may disrupt normal anti-apoptotic insulin signaling, leading to retinal cell death. Our studies included assessment of neural retina function (ERG), vascular degeneration, and Müller glial cells (which express only β1 and β2-adrenergic receptor subtypes). In the current study, we produced β2-adrenergic receptor knockout mice to examine this deletion on retinal neurons and vasculature, and to identify specific pathways through which β2-adrenergic receptor modulates insulin signaling. As predicted from our hypothesis, β2-adrenergic receptor knockout mice display certain features similar to diabetic retinopathy. In addition, loss of β2-adrenergic input resulted in an increase in TNFα, a key inhibitor of insulin receptor signaling. Increased TNFα may be associated with insulin-dependent production of the anti-apoptotic factor, Akt. Since the effects occurred *in vivo* under normal glucose conditions, we postulate that aspects of the diabetic retinopathy phenotype might be triggered by loss of β2-adrenergic receptor signaling.

## Introduction

Although diabetic retinopathy is recognized as the leading cause of blindness in working age adults, we have yet to define the cellular mechanisms responsible for diabetes-induced loss of retinal neurons. Several lines of evidence suggest a link between decreased sympathetic innervation and diabetes. For example, hyperglycemia has been shown to cause dysfunctional neurotransmitter release from the sympathetic ganglia projection to the retina [1]. In our own studies, we have previously shown that removal of the superior cervical ganglion or knockout of dopamine beta hydroxylase (a key enzyme in the conversion of dopamine to norepinephrine in sympathetic neurons) results in a retinal phenotype that is similar to that seen in diabetic animals [2], [3]. Likewise, we showed that treatment with adrenergic receptor antagonists, in particular β-adrenergic receptor antagonists, caused a similar diabetic phenotype in retina [4], [5]. These results led us to hypothesize that restoration of β-adrenergic signaling in diabetic retina might prevent or reduce retinal damage due to diabetes. To test this hypothesis, we treated streptozotocin-induced diabetic rats with a general β-adrenergic receptor agonist. As predicted, the treatment prevented retinal damage in this model system. [6], [7].

Two cell types involved in retinal changes of diabetes are retinal vascular endothelial cells (REC) and Müller glial cells, which express different subtypes of β-adrenergic receptors. REC express only β1- and β3-adrenergic receptors [8] whereas Müller cells posses β1- and β2-adrenergic receptors [9]. Our previous studies have shown that β1-adrenergic receptor knockout mice exhibit retinal changes similar to diabetic animals in spite of normal glucose levels [10]. This suggests that loss of adrenergic signaling through the β1-adrenergic receptor subtype on REC and/or Müller cells may be involved in mediating diabetic/hyperglycemic retinal damage.

β2-adrenergic receptors are also likely to play a significant role, potentially in mediating Müller cell responses to hyperglycemia [11]. β2-adrenergic receptors appear to initiate protective responses in hyperglycemic Müller cells by decreasing TNFα signaling [11], thus restoring normal insulin receptor activity and suppressing apoptosis *in vitro*. In order to further determine the specific role of β2-adrenergic receptors in retina changes *in vivo*, we now provide the first analysis of retinal changes in β2-adrenergic receptor knockout mice. We report here that β2-adrenergic receptor knockout mice exhibit attributes of retinal changes common in diabetes, despite normal glucose levels. Our results further suggest that *in vivo* loss of β2-adrenergic signaling triggers an increase in TNFα levels that leads to reduced insulin signaling and increased retinal cell apoptosis, similar to our observations *in vitro* [11]. Since, these specific alterations mimic the molecular changes seen in diabetic retinopathy, it is possible that changes in β2-adrenergic receptor signaling acts as a molecular trigger for diabetes-induced retinal damage.

## Methods

### Mice

All mice experiments, including those for dark-adaptation and tail electrodes for ERG analyses, were approved by the Institutional Animal Care and Use Committee at the University of Tennessee Health Science Center (Protocol #1992). β1/β2-adrenergic receptor knockout mice (*Adrb1^tm1^Bkk Adrb2^tm1^Bkk*/J) were purchased from Jackson laboratories (Bar Harbor, ME). C57BL6 wildtype mice were purchased from Charles River Laboratories. β1/β2-adrenergic receptor knockout mice were generated on a mixed background (129S1/Sv * 129X1/SvJ * C57BL/6J * DBA/2 * FVB/N ). We appreciate that C57BL6 may not be the ideal wildtype control, but the original β1/β2-adrenergic receptor knockout mice were from a mixed background containing C57BL6, therefore we chose the C57BL6 for wildtype. Since we use these mice at 2 months of age, other issues from the C57BL6 background should be minimized. β2-adrenergic receptor knockout mice have been previously characterized and shown to be fertile, with normal heart rate and blood pressure [12], but with a blunted hypotensive response to isoproterenol (a classic β-adrenergic receptor agonist) compared to wildtype mice [12].

Genotyping was performed by PCR analysis using genomic DNA isolated from tail snips of mice (Sigma-Aldrich). Briefly, genomic DNA is extracted from the mice tail sample that has been incubated in Tissue Preparation Solution and Extraction Solution for 10 minutes at room temperature. The sample is heated to 95°C for 3 minutes and then mixed with a neutralized solution to neutralize inhibitory substances prior to PCR. An aliquot of the DNA extract is then added directly to the optimized PCR mix supplied. PCR was carried out using GoTaq® Hot Start Polymerase kit (Promega) with the following conditions: 98°C 90 s (1 cycle); 98°C 30 s, 64°C 40 s, 72°C 50 s (2 cycles); 95°C 30 s, 64°C 40 s, 72°C 50 s (33 cycles); 72°C 5 min (1 cycle). Primers used for the analysis were as follow:

T11: GGGGCTGCTAAAGCGCATGCTCC (23) pPGK promoter of Neo^R^, anti

T36: GGGAAGACAATAGCAGGCATGCT (23) bGHpA of PGK-Neo^R^, sense

T131: CGCTATGTTGCTATCACATCGCC (23) mβ2 gene, sense

T132: GATTTGTCTATCTTCTGCAGCTGCC (25) mβ2 gene, anti

T140: GGCTCTCTACACCTTGGACTCCG (23) mβ1 gene, anti

T141: CCGCCGCCGTCCCCCGG (17) mβ1 gene, sense

**Figure 1:**
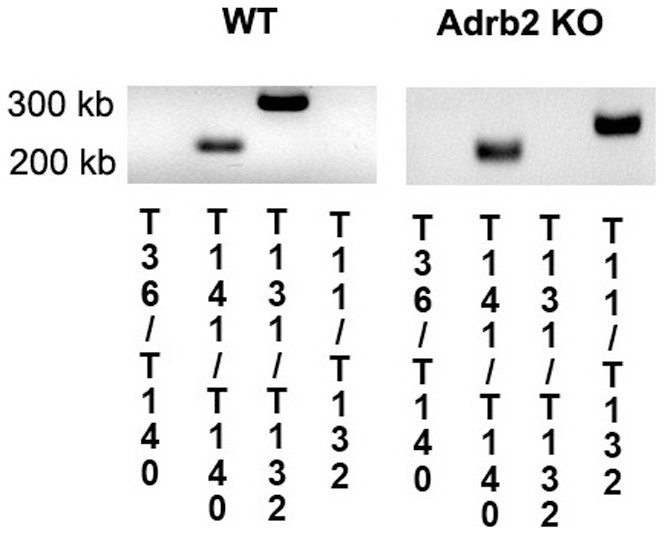
Genotyping results. Results of the genotyping to verify that the β2-adrenergic receptor is eliminated in the KO mice. Numbers on the bottom correspond to primers described in the methods to demonstrate effective β2-adrenergic receptor knockout.

The mice used for the experiment were sacrificed and ocular tissues collected at 2 months of age. PCR images are presented as Figure 1.

### Electroretinogram (ERG)

Prior to sacrifice for morphological and biochemical analyses, animals were subjected to ERG analyses to evaluate the changes in the electrical activity of the retina as we have done previously [5], [7]. Briefly, mice were dark-adapted overnight. ERG responses were recorded from both eyes together using platinum wire corneal electrodes, forehead reference electrode, and ground electrode in the tail. Pupils were fully dilated using 1% tropicamide solution (Alcon). Methylcellulose (Celluvise; Allergan, Irvine, CA) drops were applied as well to maintain a good electrical connection and body temperature was maintained at 37°C by a water-based heating pad. All ERG experiments were approved by the University of Tennessee Institutional Animal Care and Use Committee on Protocol #1992. ERG waveforms were recorded with a bandwidth of 0.3–500 Hz and samples at 2 kHz by a digital acquisition system and were analyzed a custom-built program (MatLab). Statistics was done on the mean ±SD amplitudes of the a- and b- wave of each treatment group.

### Neuronal Analyses

Formalin-fixed paraffin sections were stained with hematoxylin and eosin for light microscopy and morphometry of retinal thickness as previously described [6]. Photomicrographs were assessed for retinal thickness and the number of cells in the ganglion cell layer using methods previously described. The thickness of the retina and the cell count were measured using OpenLab software (Improvision, Lexington, MA) [7].

### Vascular Analyses

Retinas from 1 eye of wildtype and β2-adrenergic receptor knockout mice were used to count degenerate capillaries. The eyes were enucleated, suspended in 10% buffered formalin for 5 days, and the retina was dissected in 3% crude trypsin solution (Difco Bacto Trypsin 250, Detroit, MI). The retinal vascular tree was dried onto a glass slide and stained with hematoxylin-periodic acid-Shiff. Degenerate capillaries were counted and identified as previously described [7].

### Western Blot Analysis

Equal amounts of protein from the tissue extracts were separated on the pre-cast tris-glycine gel (Invitrogen, Carlsbad, CA), blotted onto a nitrocellulose membrane. After blocking in TBST (10 mM Tris-HCl buffer, pH 8.0, 150 mM NaCl, 0.1% Tween 20) and 5% (w/v) BSA, the membrane was treated with appropriate primary antibodies followed by incubation with secondary antibodies labeled with horseradish peroxidase. Antigen-antibody complexes were detected by chemilluminescence reagent kit (Thermo Scientific). Primary antibodies used were phosphorylated Akt (Serine 473), Akt, Cytochrome C, Bax, Bcl-xL, SOCS3, GFAP, phosphorylated insulin receptor (tyrosine 1150/1151), insulin receptor (all purchased from Cell Signaling, Danvers, MA), and beta actin (Santa Cruz).

### ELISA Analysis

A cleaved caspase 3 ELISA (Cell Signaling, Danvers, MA) was used to measure levels of the active apoptotic marker in whole retinal lysates. TNFα protein concentrations were measured using a TNFα ELISA (ThermoFisher, Pittsburgh, PA). For cleaved caspase 3 ELISA analyses, equal protein was loaded (50 ug) into all wells to allow for comparisons based on O.D. For the TNFα ELISA, 50 ug protein was loaded into all wells, with analyses based on a standard curve.

### Terminal Deoxynucleotidyl Transferase Mediated dUTP Nick End Labeling Assay (TUNEL)

TUNEL analyses were completed on C57/BL6 mice and β2-adrenergic receptor knockout mice at 2 months of age. TUNEL was done according to manufacturer’s instructions using the ApopTag-Red kit (Millipore, Bilerica, MA).

### Immunohistochemical Staining

β2-adrenergic receptor KO and C57/BL6 wildtype mice at 2 months of age were perfused, under deep Avertin anesthesia, with 4% paraformaldehyde for 15 min. Eyes were then removed, followed by a 30 min immersion in the same fixative. Ten μm cryostat sections contained retinas were collected and underwent double immunofluorescent staining. Briefly, the sections were incubated in 1% bovine serum albumin and 0.3% Triton X-100 (Sigma) in PBS for 1 h at room temperature, followed by incubation with rabbit anti-GFAP and mouse anti-insulin receptor, or mouse anti-GFAP and rabbit anti TNFα (all primary antibodies were purchased from Abcam, MA) overnight at 4°C. Following rinses in PBS, sections were incubated with Alexa Fluor 594 or Alexa Fluor 488 secondary antibody for 1 h at room temperature. Sections were counterstained with DAPI (1 μM, Invitrogen,Carlsbad, CA) to label all cell nuclei for 3 min at room temperature. Sections were observed and analysis using a Zeiss710 Confocal microscope.

### Statistics

For all analyses, all experiments were done in triplicate. Data is presented as mean ± SEM, with statistical analyses using Kruskal-Wallis non-parametric testing, followed by Dunn’s test. For Western blot analyses, data was normalized to beta actin levels.

## Results

Previous studies in various mouse models of diabetic retinopathy have established four major functional and morphological markers that help to define the disease. Retinas from β2-adrenergic receptor KO mice displayed three of the four markers as described below.

### Marker #1

#### B-wave and oscillatory potential amplitudes were reduced in β2-adrenergic receptor KO mice compared to wild type at 2 months of age

**Figure 2:**
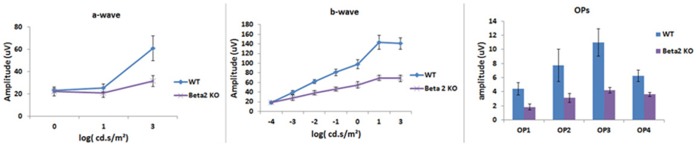
ERG levels. Mean ERG amplitudes for the β2-adrenergic receptor knockout and wildtype mice. Left panel is the A-wave, middle panel represents the B-wave, and the right panel is the amplitudes for the oscillatory potentials. N = 6 mice in each group.

At all light intensities evaluated, the β2-adrenergic receptor knockout mice had reduced B-wave (Figure 2 middle) and oscillatory potential (Figure 2 right) amplitudes. A-wave responses of KO and wild type were statistically significantly different at high light intensity.

### Marker # 2

#### β2-adrenergic receptor knockout mice had thinner retinas overall and fewer cells in the ganglion cell layer

**Figure 3:**
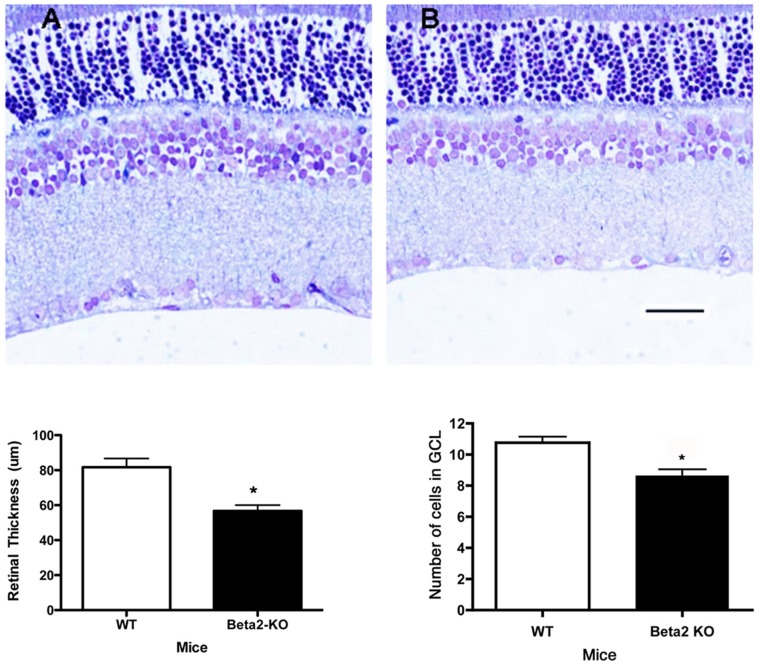
Neuronal Changes. Panel A shows mean retinal thickness in β2-adrenergic receptor knockout and wildtype mice. Panel B shows the cell numbers in the ganglion cell layer. A representative image is provided. *P<0.05 vs. wildtype mice. N = 6 mice in each group. Scale bar is 20 um.D.

We measured the retinal thickness and cell number in the ganglion layer at 2 months of age in the β2-adrenergic receptor knockout mice. The knockout mice had significantly decreased retinal thickness and cell number in the ganglion cell layer compared to wild type (Figure 3).

**Figure 4:**
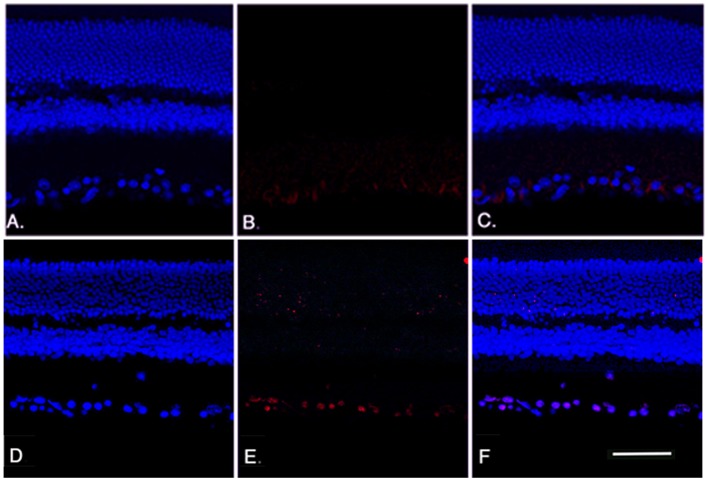
Apoptosis staining analyses. TUNEL results for wildtype mice (A-C) and β2-adrenergic receptor KO mice (D-F) showing DAPI staining (A, D) and TUNEL labeling (B, E) with overlay provided in panels C, F. Scale bar is 50 um.

The loss of cells in the ganglion cell layer was due to apoptosis, reflected in an increase in TUNEL labeling in the ganglion cell layer of the β2-adrenergic receptor knockout mice. We performed TUNEL labeling on retinal sections to measure cell death in the β2-adrenergic receptor knockout mice. Figure 4 (Panel E) shows increased TUNEL-labeling in the ganglion cell layer of β2-adrenergic receptor knockout mice compared to their wildtype littermates (Panel B).

**Figure 5:**
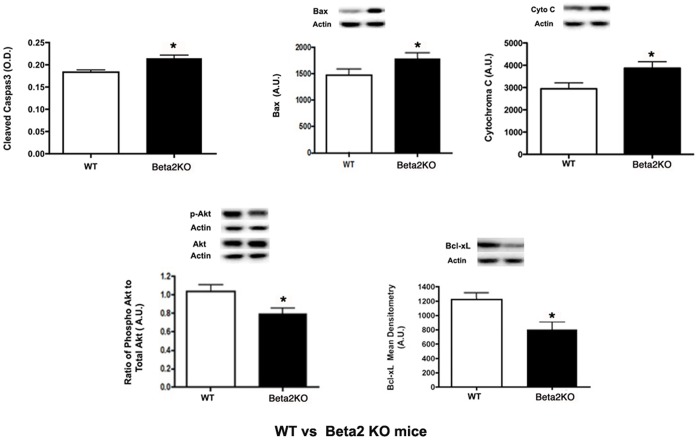
Apoptotic protein levels. Cleaved caspase 3 ELISA and Western blot results for wildtype mice and β2-adrenergic receptor KO mice for pro-apoptotic proteins (top panel-cleaved caspase 3, Bax, Cytochrome C) and anti-apoptotic proteins (bottom panel-phosphorylated Akt, Bcl-xL). A representative Western blot is provided. All Western blot data were normalized to beta actin levels. ELISA data was normalized to protein loaded into well. *P<0.05 vs. wildtype. N = 6 mice in each group.

In addition to TUNEL labeling, we used protein analyses of whole retinal lysates from the wildtype and β2-adrenergic receptor knockout mice for key pro- and anti-apoptotic proteins. We found that all pro-apoptotic proteins evaluated (cleaved caspase 3, bax, cytochrome C) were increased in the β2-adrenergic receptor knockout mice (Figure 5, top panels), while the anti-apoptotic proteins (phosphorylated Akt and Bcl-xL) were significantly reduced (Figure 5, bottom panels). The altered levels of these proteins is likely involved in the decreased ERG signal from the inner retina (B-wave), as well as the increased TUNEL labeling.

### Marker # 3

#### Increased GFAP labeling and protein levels in β2-adrenergic receptor knockout mice

**Figure 6:**
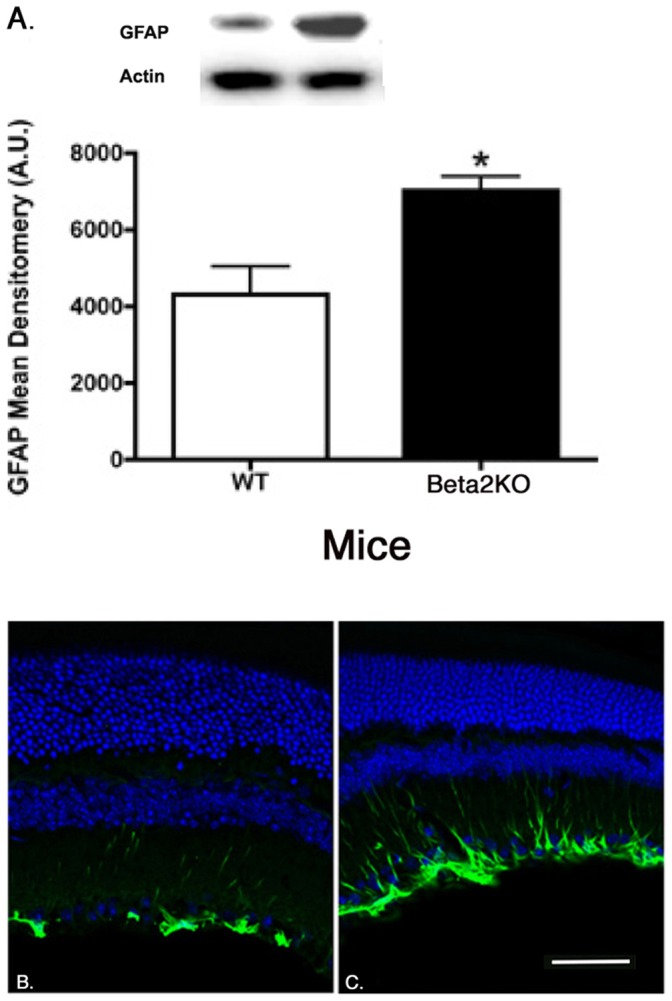
Müller cell staining for GFAP. Western blot results (A) and GFAP labeling in wildtype (B) and β2-adrenergic receptor knockout mice (C). *P<0.05 vs. wildtype. N = 5 for Western blot results.

We have previously published that β2-adrenergic receptors are critical in insulin receptor signaling in retinal Müller cells [11]. To evaluate whether retinal Müller cells were activated in the β2-adrenergic receptor knockout mice, we measured protein levels of GFAP and performed immunofluorescent staining for GFAP, which is increased in retinal Müller cells once activated [13]. We found that GFAP protein levels were significantly increased in the β2-adrenergic receptor knockout mice compared to wildtype (Figure 6).

### Marker # 4

#### Retinas from 2 month old β2-adrenergic receptor KO mice did not show the increase in degenerate capillary numbers normally associated with the diabetic phenotype

**Figure 7:**
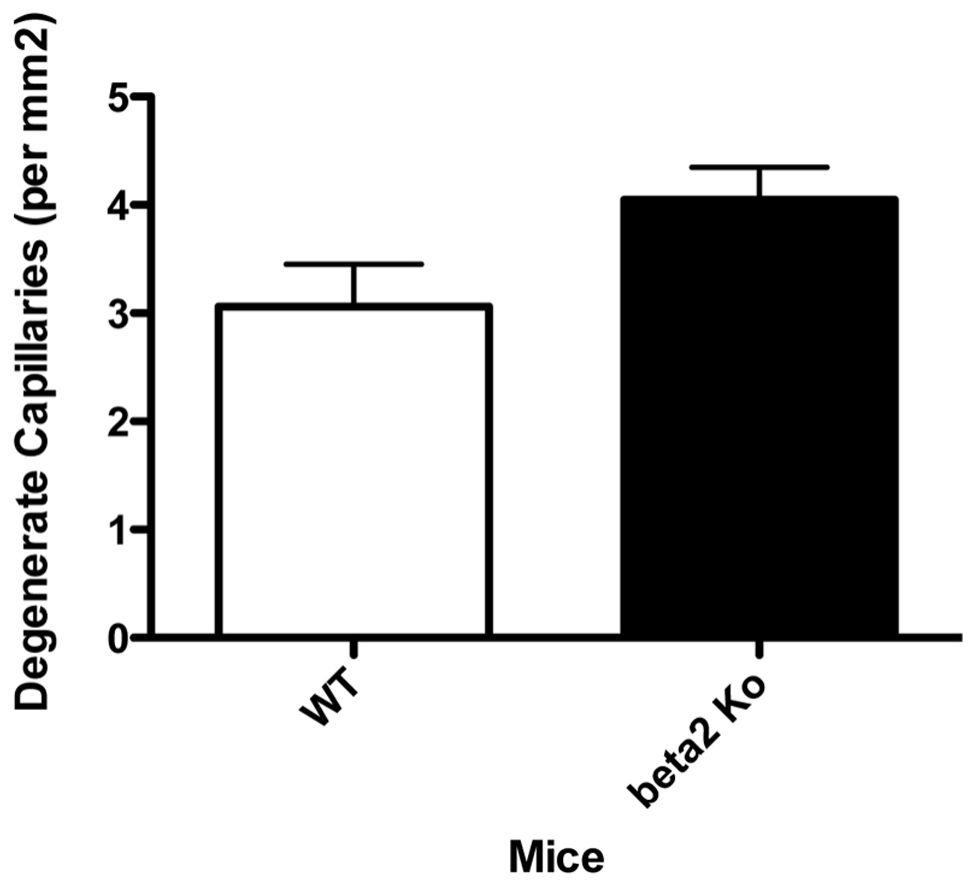
Vascular Analyses. Panel A shows degenerate capillary numbers in β2-adrenergic receptor knockout mice and wildtype mice. N = 5 mice in each group.

We measured the numbers of degenerate capillaries in the β2-adrenergic receptor KO mice at 2 months of age and found a trend towards increased capillary degeneration in the receptor knockout animals, but this did not achieve statistical significance versus that seen in wild type retinas. (Figure 7). We have previously shown increases in degenerate capillaries in β1-adrenergic receptor knockout mice [10] and animals treated with streptozotocin to induce diabetes [6], [7]; however, these changes were not observed until 4–6 months of age or 6 months after treatment. Thus the assessment in β2-adrenergic receptor KO mice at 2 months may not have allowed sufficient time for degenerate capillaries to form. Experiments are now underway to repeat these experiments using KO mice at 6 months of age.

### Insulin Receptor Phosphorylation was Reduced in β2-adrenergic Receptor Knockout Mice

**Figure 8:**
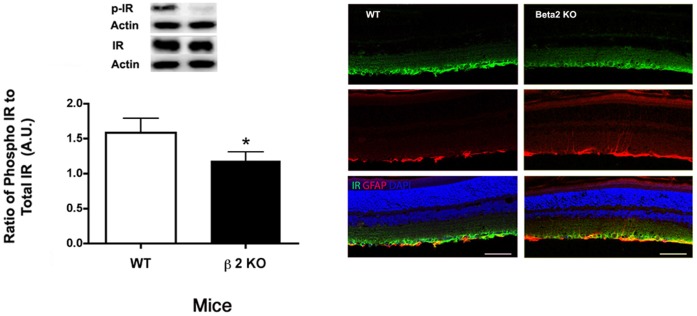
Insulin Receptor Levels and localization. Left Panel: Western blot results for phosphorylated insulin receptor (Tyr 1150/1151) in β2-adrenergic receptor knockout mice vs. wildtype. Western blot data were normalized to beta actin. N = 5 mice in each group. *P<0.05 vs. wildtype. Right Panel: Confocal microscopy with co-labeling for insulin receptor (green), GFAP (red), nuclei (blue). Scale bar is 50 um.

We have previously reported that hyperglycemia significantly reduced insulin receptor phosphorylation on tyrosine 1150/1151, which was restored following treatment with a β-adrenergic receptor agonist, Compound 49b [7]. In retinal Müller cells, we reported that insulin receptor signaling was altered when cells were grown in high glucose, which led to increased apoptosis that was prevented when Müller cells were treated with the β2-adrenergic receptor agonist, salmeterol [11]. To confirm those studies *in vivo*, we measured insulin receptor phosphorylation in the β2-adrenergic receptor knockout mice. As predicted, receptor phosphorylation was significantly reduced (Figure 8), and the decrease was specifically associated with retinal Müller cells based on immunoflourescent staining of the receptor that co-localized with GFAP-labeled Muller cells.

### TNFα was Increased in β2-adrenergic Receptor KO Mice

**Figure 9:**
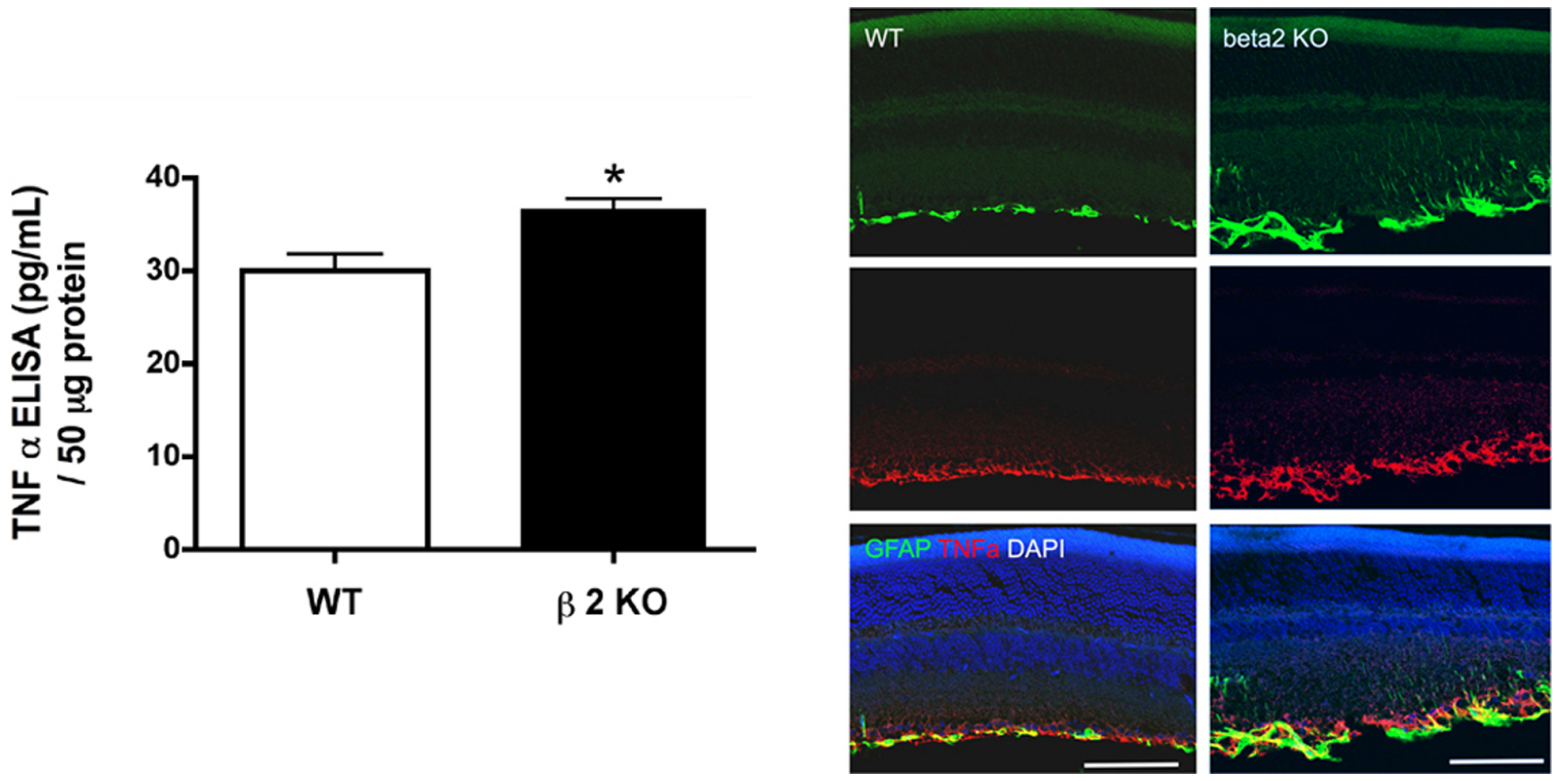
TNFα levels and localization. Top Left Panel: TNFα concentrations in retinal lysates from β2-adrenergic receptor knockout and wildtype mice. *P<0.05 vs. wildtype. ELISA data was normalized to protein loaded into well. N = 6 mice in each group. Top Right Panel: Confocal microscopy for TNFα (red), GFAP (green), and nuclei (blue). Scale bar is 50 um.

In retinal endothelial cells, we have shown that TNFα can inhibit insulin receptor signaling through activation of suppressor of cytokine 3 (SOCS3), coincident with increased retinal endothelial cell apoptosis [14]. We found that loss of β2-adrenergic receptor signaling triggering significant increases in TNFα (Figure 9) This suggests that loss of β2-adrenergic receptor signaling changes cellular signaling in a manner similar to that seen in the hyperglycemic state.

## Discussion

### Loss of β2-adrenergic Receptor Expression Results in Neuroglial Abnormalities of Common to Diabetic Retinopathy

A major finding from these studies is that β2-adrenergic receptor knockout mice display neuronal and functional markers similar to those observed in diabetic mice, despite the presence of normal levels of glucose. This is consistent with our hypothesis that loss of β2-adrenergic receptor input is a major trigger for the retinal changes observed in diabetes. Since retinal β2-adrenergic receptors are primarily localized to Müller cells, our findings also highlight the specific role Müller cells play in this blinding disorder. Similar to other glial cells in CNS, Müller cells display increased GFAP labeling in response to various types of stress. We find that in our KO model, GFAP expression is increased in Müller cells, indicative of a direct stress response to the loss of input through their β2-adrenergic receptors.

### TNFα is Increased in β2-adrenergic Receptor KO Mice

Since we have previously reported that β-2-adrenergic receptors can regulate TNFα levels [9], [11], we measured retinal TNFα levels in retinal lysates from β2-adrenergic receptor knockout mice. Our results show a significant increase in overall levels and, based on immunofluorescence studies, the increase is largely localized to Müller cells. Our results show for the first time TNFα levels are increased in retinal lysates from β2-adrenergic receptor knockout mice. The mechanisms and specific cell types involved in the TNFα pathways are currently under investigation.

### β2-adrenergic Receptor Signaling Helps Maintains Insulin Receptor Signaling

Retinal Müller cells undergo increased apoptosis when cultured in high glucose conditions that are known to lead to insulin resistance [15], Our previous work *in vitro* has suggested that a functional link may exist between maintenance of β2-adrenergic receptor function and maintenance of insulin receptor anti-apoptotic pathways. We observed that decreased insulin receptor signaling could subsequently be reversed by restoring β-2-adrenergic receptor stimulation [11]. We also showed that the opposite is true: loss of β2-adrenergic receptor stimulation leads to decreased insulin receptor signaling. This agrees with the current *in vivo* findings, as insulin receptor phosphorylation is reduced in the β2-adrenergic receptor knockout mice.

### β2-adrenergic Receptor Signaling Prevents Expression of Inhibitors of Insulin-dependent Pro-apoptotic Pathways

Our results demonstrate that TNFα a negative regulators of the insulin receptor pathway is increased in the absence β2-adrenergic receptor input. Once produced, these negative regulators can block the insulin-induced activation/phosphorylation of a key apoptotic factor, Akt. Thus, we hypothesize that the diabetes-induced, systemic loss of β2-adrenergic receptor input to retinal cells, causes further damage to the retina by triggering production of TNFα, which in turn disables the anti-apoptotic actions of insulin receptor pathways. Loss of insulin signaling through insulin resistance, loss of β2-adrenergic receptor input, and production of the anti-apoptotic inhibitors, TNFα, could lead to retinal cell death associated with retinopathy.

In conclusion, absence of β2-adrenegic receptor input *in vivo* results in morphological and function damage to the retina including retinal thinning, cell loss in the retinal ganglion cell layer, and a decrease in A-wave, B-wave and oscillatory potential amplitudes - all markers associated with diabetes in rodents. We do want to state that we cannot completely rule out that some of the observed differences between the two groups could be due to strain-dependent differences, since we used only the C57/B6 background for the wildtype mice. These findings establish β2-adrenergic receptor agonists as promising candidates for drug therapy to prevent retinal cell apoptosis, potentially acting on Müller cell pathways to maintain insulin signaling and homeostasis of the retina. We have recently reported that treatment with a Compound 49b, a novel β-adrenergic receptor agonist, significantly increases insulin receptor phosphorylation, while decreasing TNFα and retinal cell apoptosis [7]. Future studies will focus on further elucidating the actions of Compound 49b, as well as its potential for treatment of early stage diabetic retinopathy.
